# Interactions between Silicon Oxide Nanoparticles (SONPs) and U(VI) Contaminations: Effects of pH, Temperature and Natural Organic Matters

**DOI:** 10.1371/journal.pone.0149632

**Published:** 2016-03-01

**Authors:** Hanyu Wu, Ping Li, Duoqiang Pan, Zhuoxin Yin, Qiaohui Fan, Wangsuo Wu

**Affiliations:** 1 Radiochemistry Laboratory, School of Nuclear Science and Technology, Lanzhou University, Lanzhou, Gansu, 730000, China; 2 Key Laboratory of Petroleum Resources, Gansu Province / CAS Key Laboratory of Petroleum Resources Research, Institute of Geology and Geophysics, Chinese Academy of Sciences, Lanzhou, Gansu, 730000, China; 3 Institute of Nuclear Physics and Chemistry, China Academy of Engineering Physics, Mianyang, Sichuan, 621000, China; 4 Key Laboratory of Special Function Materials and Structure Design, Ministry of Education, Lanzhou, Gansu, 730000, China; University of Helsinki, FINLAND

## Abstract

The interactions between contaminations of U(VI) and silicon oxide nanoparticles (SONPs), both of which have been widely used in modern industry and induced serious environmental challenge due to their high mobility, bioavailability, and toxicity, were studied under different environmental conditions such as pH, temperature, and natural organic matters (NOMs) by using both batch and spectroscopic approaches. The results showed that the accumulation process, i.e., sorption, of U(VI) on SONPs was strongly dependent on pH and ionic strength, demonstrating that possible outer- and/or inner-sphere complexes were controlling the sorption process of U(VI) on SONPs in the observed pH range. Humic acid (HA), one dominated component of NOMs, bounded SONPs can enhance U(VI) sorption below pH~4.5, whereas restrain at high pH range. The reversible sorption of U(VI) on SONPs possibly indicated that the outer-sphere complexes were prevalent at pH 5. However, an irreversible interaction of U(VI) was observed in the presence of HA (Fig 1). It was mainly due to the ternary SONPs-HA-U(VI) complexes (Type A Complexes). After SONPs adsorbed U(VI), the particle size in suspension was apparently increased from ~240 nm to ~350 nm. These results showed that toxicity of both SONPs and U(VI) will decrease to some extent after the interaction in the environment. These findings are key for providing useful information on the possible mutual interactions among different contaminants in the environment.

## Introduction

One of the stern challenges faced by the world is the potential contamination of the biosphere, lithosphere, and underground hydrosphere with heavy metal ions, radionuclides, and nanomaterial. Uranium is known as a main nuclear hazard in the nuclear industry, and its sorption behaviors at solid/water interface such as clay, mineral, oxide, soil, and nanomaterial play a very important role in controlling the transportation and migration of uranium in environment [1–5]. During the past decades, the sorption of U(VI) has been widely studied at micro- and macro-scales using batch, surface complexation model and spectroscopies [4–6]. It is well known that U(VI) mainly presents as UO_2_^2+^, hydrolysis, organic-U(VI) complexes, and soluble carbonates in aqueous solution [7]. The interaction between these species of U(VI) and adsorbent with high surface area is kinetically fast and stable at a wide range of pH. A general knowledge can be drawn that the sorption of U(VI) at the solid/water interface could alleviate the chemical toxicity of uranium aggravating health concerns [8,9].

**Fig 1:**
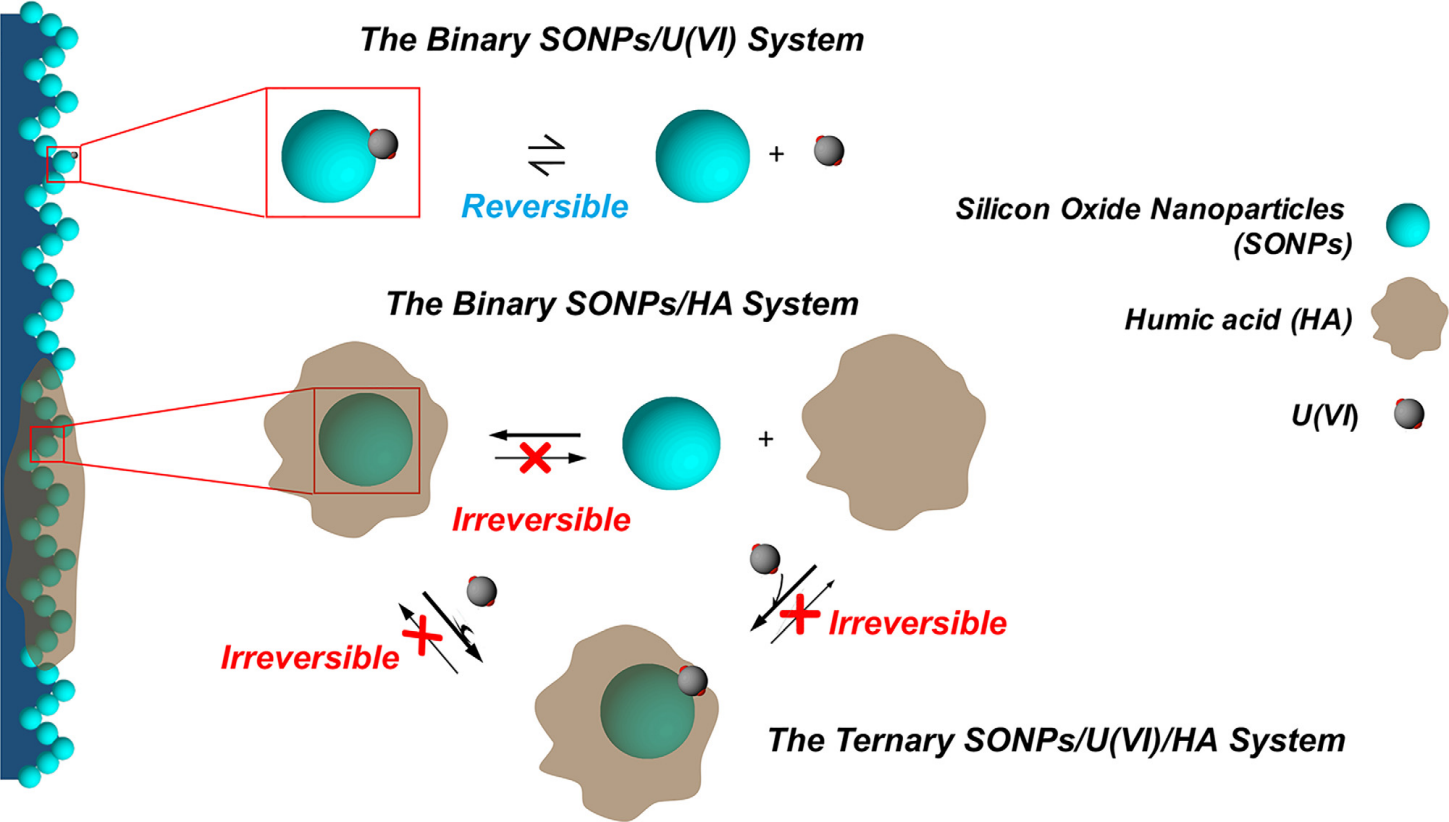
Binding modes and reversibility of interactions among SONPs, U(VI), and HA. The interactions of different systems occur in the aqueous phase. Blue particle presents SONPs; Gray particle presents U(VI); Brown part presents HA.

Nanomaterial has been utilized increasingly and widely in different fields including medicine, pharmaceutical, food, energy, and engineering due to its excellent properties. On the other hand, nanomaterial also bears a high potential risk of inevitably being released into the environment, which can raise very high challenge to the biosphere in terms of high toxicity. Nowadays, the possible behaviors of nanomaterial contaminations in environment have already attracted increasing more and more worldwide attentions [10]. Silicon dioxide nanoparticles (SONPs), one of the most-used nanomaterial in the modern industry, have been proven toxic to antimicrobial activity and soil microbial communities [11,12]. It has been reported that SONPs can affect human being in vitro/vivo [13–16]. Napierska et al. [15] illustrated that SONPs (14, 15 and 16 nm in diameter) can cause cytotoxic damage in human endothelial cells. McCarthy et al. [14] also confirmed that SONPs (~10 nm) have high toxicity on human being such as inflammation, apoptosis, and decreasing survival lung submucosal cells, but the large size of SONPs with 150 and 500 nm exerted no obvious toxic effects. Moreover, amorphous SONPs also have effects on bacteria, yeast, algae, fish, and even the whole ecological environment [12,16].

Natural and synthetic silicon oxide exists extensively in environment and is applied in efficacious removal of heavy metals/radionuclides for several years [17–19]. Generally, the transportation of contaminations in environment depends on the existed contaminations types and the reversibility between contaminations [20]. Therefore, one might be speculated that employing waste SONPs discharged from industry as adsorbent in sequestrating U(VI), which can reduce the hazardous risk from both U(VI) and SONPs when two different types of contaminations co-exist in the environment and develop the utilization of waste SONPs as adsorbent.

Furthermore, natural organic matters (NOMs) play an environmentally significant role in interaction properties between adsorbents and heavy metals/radionuclides, such as free-ion immobilization, biological availability and transportation [21]. Humic acid (HA), as one dominant ingredient of NOMs, has a variety of functional groups which could complex with both metal ions and nanomaterial. These interactions would not only alter the environmental fate of nanomaterial, but also affect the removal and transportation of heavy metals/radionuclides by nanomaterial [22]. To the best of our knowledge, research on the interactions between different contaminations such as waste SONPs and U(VI) is still scarce; however, it is very important to understand the environmental fate of contaminants and the interaction mechanism among contaminations. The present work is aimed to identify the interaction between U(VI) and SONPs under environmental conditions such as pH, ionic strength, temperature, and HA used as an analogue of NOMs.

## Experimental

### Materials and Reagents

All chemicals were purchased as analytically pure and used without any further purification. SONPs were prepared as colloidal suspension. U(VI) stock solution was prepared by dissolving uranyl nitrate hexahydrate (UO_2_(NO_3_)_2_∙6H_2_O) in Milli-Q water and kept at pH 3.0 by adding negligible hydrogen nitrate. HA, one of the most important NOMs in nature, extracted from Lintan County (Gansu, China) soil had been characterized previously [23].

### Batch Sorption

All batch sorption experiments were carried out at ambient conditions. The particular amount of stock solution of NaClO_4_, U(VI) and SONPs suspension were added in 10 mL polyethylene test tubes to achieve the desired concentrations of each component, and the total volume of sorption system was maintained at 6.0 mL. The pH value of each sorption system was adjusted by adding the negligible HClO_4_ and/or NaOH solution, due to the usage of NaClO_4_ controlling ionic strength and each sorption system is in equilibrium with atmospheric air. After the suspensions were shaken at 298 K for 24 h except for kinetic experiments, the solid and liquid phases were separated by centrifugation at 12,000 rpm for 30 min. The concentration of U(VI) in the supernatant (*C*_e_) was analyzed by spectrophotometry at a wavelength of 652 nm using Arsenazo III. The sorption of HA followed the U(VI) sorption procedures mentioned above, and the concentration of HA in the supernatant was determined with an UV-*vis* spectrophotometer. For the thermodynamic experiments, a series of U(VI) concentration was applied at different shaking temperatures of 298, 318 and 338 K. For the desorption experiments, half of the supernatant was replaced by an equal volume of background electrolyte solution with same pH value after complete sorption, and the desorption system was shaken for 168 or 504 h and then separated and analyzed as mentioned before.

All the experimental data were the averages of duplicate or triplicate experiments, and the relative errors of the data were less than 5%.

The percent sorption (%) of U(VI) was calculated as follows:

$$\text{Sorption}\;(\%)=\frac{C_{0}-C_{e}}{C_{0}}\times 100\%$$ (1)

where *C*_0_ (mol/L) and *C*_e_ (mol/L) are the initially added, and equilibrium or supernatant (for kinetic experiments) concentrations of U(VI), respectively.

### Characterization Studies

Samples were characterized by X-ray diffraction (XRD), Brunauer-Emmett-Teller (BET) surface area analysis, scanning electron microscope (SEM), transmission electron microscope (TEM), Fourier transform infrared (FT-IR) spectroscopy, and dynamic light scattering (DLS).

The XRD pattern of the composite’s structure was obtained with an X′ Pert pro Panalytical equipped with a rotation anode using Cu-*Kα* radiation, operating at 40 kV and 30 mA. The scanning angle started from 3° to 65° with a step interval of 0.02° at a rate of 1.0^o^/min. BET surface area was measured using a Micrometrics ASAP 2020 Accelerated Surface Area. SEM analysis of pre-dried samples was obtained using a Hitachi S-4800 field emission scanning electron microscope. Prior to TEM analysis, the solid samples were sprinkled onto adhesive carbon tapes supported on metallic disks, then the images were collected on a Tecnai-G2-F30 (FEI, USA) transmission electron microscope using an accelerating voltage of 300 kV. For FT-IR spectroscopy, the samples were analyzed using a Nicolet Nexus 670 Fourier transform infrared spectrometer with potassium bromide pellets. For DLS measurements, suspensions of SONPs (10 mg/L) were prepared in the absence or presence of HA (15 and 50 mg/L) after the U(VI) sorption experiments as mentioned above. Hydrodynamic diameter was determined by dynamic and electrophoretic light scattering using Malvern Zetasizer Nano ZS.

## Results and Discussion

### Characterization

**Fig 2:**
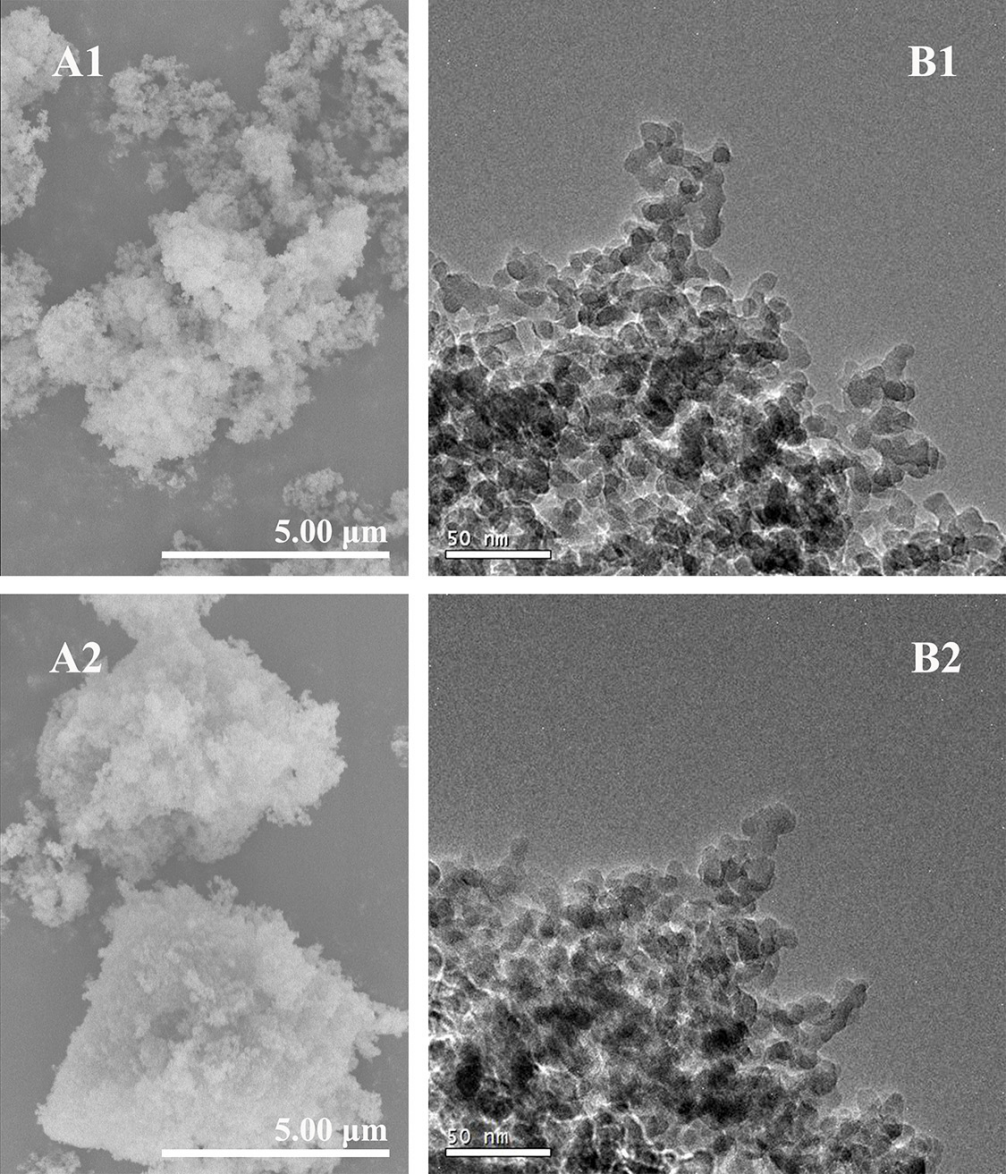
Characterization images. SEM images of (A1) SONPs, and (A2) HA-SONPs, *pH* = 3. TEM images of (B1) SONPs, and (B2) HA-SONPs, *pH* = 3.

SONPs were examined in order to ascertain the physic-chemical properties, such as crystallinity and purity, morphological features, and surface charge. XRD pattern clearly showed a single peak centered at 23 2-Theta with no evidence for the presence of any crystalline phases (i.e., amorphous characteristic of silica) or any other impurity (S1 Fig). SEM and TEM images showed that SONPs exhibited a smooth and spherical shape with around 20 nm in size (Fig 2), and it has been confirmed from the high specific surface area of SONPs about 229.59 m^2^/g measured by N_2_-BET method. It suggested that SONPs possibly possess high sorption capacity and strong affinity to metal ions as an example of U(VI). In comparison with SONPs, HA bounded SONPs became more aggregative and agglomerative (Fig 2A2 and 2B2), suggesting that the presence of HA can change the topology and morphology to some extent. Therefore, such processes could possibly induce the mutual interaction changes between U(VI) and SONPs.

### Kinetic Estimation

**Fig 3:**
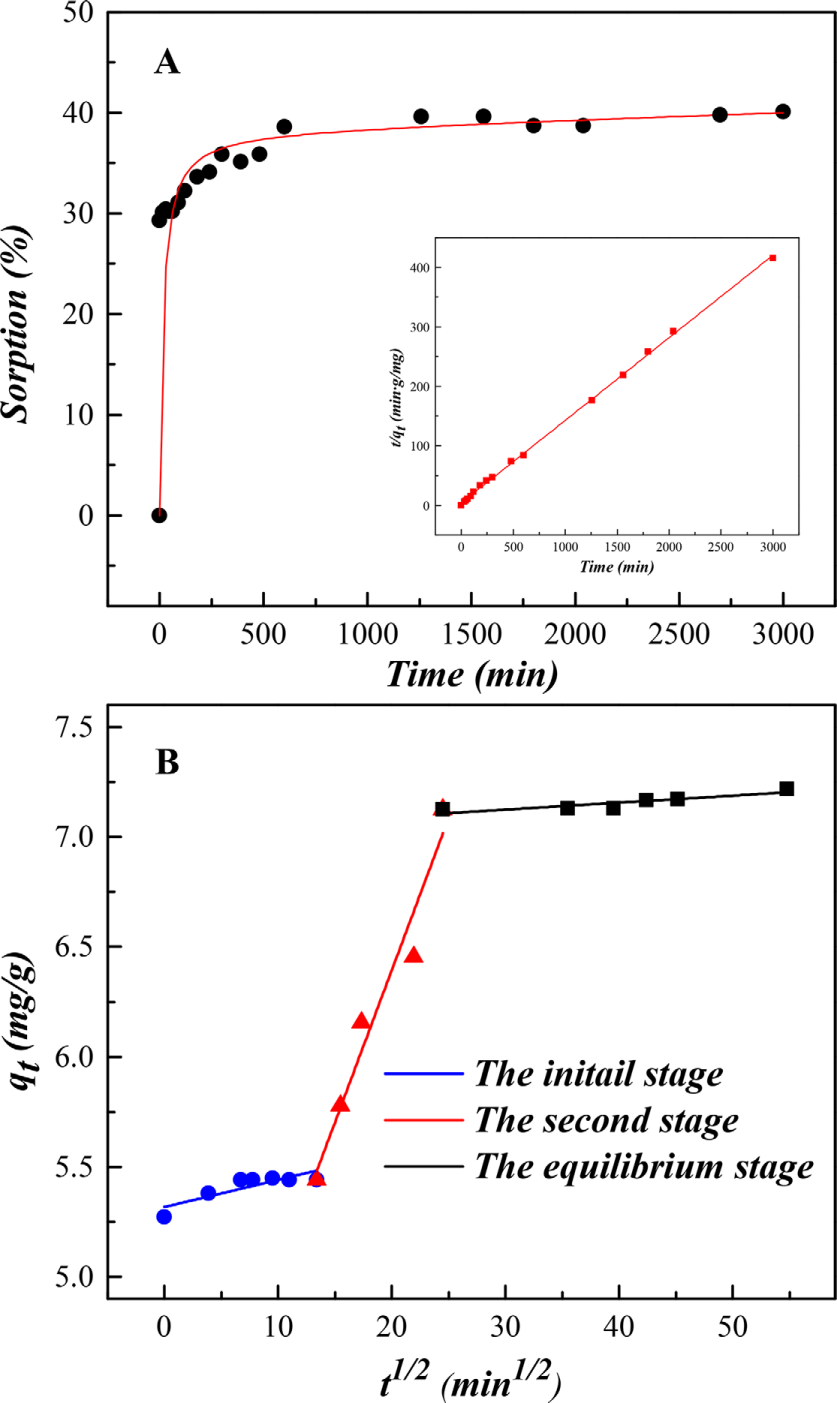
The kinetics of U(VI) sorption on SONPs (*T* = 298±1 K, *I* = 0.01 mol/L NaClO_4_, *s*/*l* = 0.6 g/L, [U(VI)] = 2.0×10^−5^ mol/L, *pH* = 5.0±0.1). (A) The effect of contact time (bottom right: The fitting plot of the pseudo-second-order equation). (B) The fitting plot of the Weber-Morris model.

The kinetics of U(VI) sorption on SONPs as a function of contact time is shown in Fig 3. U(VI) sorption achieved equilibrium within 10 h, therefore a contact time of 24 h was selected for the following experiments to ensure the equilibrium of U(VI) sorption on SONPs.

Knowledge of kinetics is important for the elucidation of sorption mechanisms. Three types of kinetic models (i.e., the pseudo-first order model, pseudo-second order model and Weber-Morris model) were employed to simulate the interaction, and the relative parameters of each model were summarized in Table 1. One can see that the correlation coefficient of the pseudo-second order model was much closer to unity (*R*^2^ = 0.9993) in comparison with the pseudo-first order model (*R*^2^ = 0.7036). This result suggested that the process can be well described by the pseudo-second order model, and U(VI) sorption can possibly be affected by both adsorbent dose and U(VI) concentration. In addition, the pseudo-second order model assumed that the controlling step might be attributed to chemical sorption or chemisorption involving valence force through sharing or exchange of electrons between two contaminations [24,25].

**Table 1:** The Kinetic Constants of U(VI) Sorption on SONPs.

Model	Equation	Parameters		
***Pseudo-first-order model***	$\frac{1}{q_{t}}=\frac{k_{1}}{q_{e}}\cdot \frac{1}{t}+\frac{1}{q_{e}}$	*q*_e_ = 6.73 mol/g	*k*_1_ = 12.49 h^-1^	*R*^2^ = 0.7036
***Pseudo-second-order model***	$\frac{t}{q_{t}}=\frac{1}{q_{e}}\cdot t+\frac{1}{k_{2}q_{e}{}^{2}}$	*q*_e_ = 7.20 mol/g	*k*_2_ = 0.0047 g/mol·h	*R*^2^ = 0.9993
***Weber-Morris model***	*q*_*t*_ = *k*_*id*_*t*^1/2^ + *C*	*k*_id,1_ = 0.0124 mg/g·min^-1/2^	*C* = 5.32	*R*^2^ = 0.6795
		*k*_id,2_ = 0.1382 mg/g·min^-1/2^	*C* = 3.63	*R*^2^ = 0.9423
		*k*_id,3_ = 0.0032 mg/g·min^-1/2^	*C* = 7.03	*R*^2^ = 0.7461

The empirically functional relation Weber-Morris plot described that adsorbate uptake varies almost proportionally with *t*^1/2^ rather than the contact time in sorption process [26]. According to the equation in Table 1, the plot would be linear and pass through the origin when the intraparticle diffusion is the unique rate-limiting step. However, it is not always the case and sorption kinetics may be controlled by film diffusion, intraparticle diffusion or other mechanism simultaneously. Thus, the plot would be multi-linear and the intercept would not equal to zero [25,27].

From Fig 3B, the multi-linear plots, which do not pass through the origin, indicated that more than one mechanism might control the sorption process of U(VI). An initial gentle-sloped portion followed by a steep-sloped linear portion and then a plateau at equilibrium illustrated that the sorption was a continuous and stepwise process. The initial stage (from 0 to 3 h) was attributed to the exterior boundary layer diffusion or instantaneous sorption of the most readily available adsorbing sites on SONPs surface [28,29], while the steep second stage (from 3 to 10 h) can be ascribed to the interior boundary layer diffusion. It should be noted that at *t* > 10 h, the sorption started to slow down as U(VI) concentration decreased in aqueous phase and then reached the final equilibrium [28,30]. The parameters calculated are listed in Table 1, where the rate constants (*k*_id,1_, *k*_id,2_ and *k*_id,3_) can be attributed to the sorption stages of the exterior layer, interior layer and equilibrium, respectively. The increasing values of *C* (μg/g) indicated that U(VI) sorption on SONPs were less influenced by the boundary layer thickness [27,29].

### Effects of pH and U(VI) Concentration

**Fig 4:**
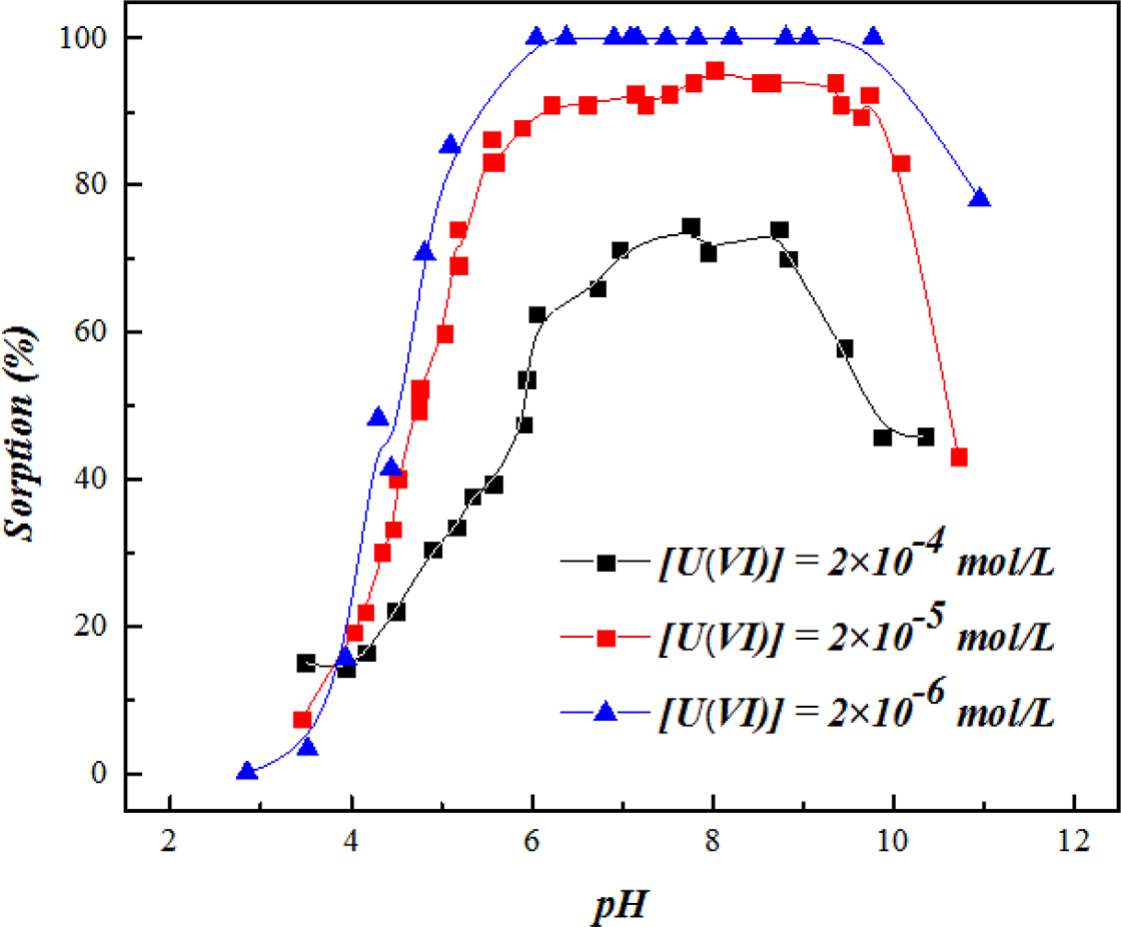
The influence of pH on U(VI) sorption on SONPs under varying uranium concentrations (*T* = 298±1 K, *I* = 0.01 mol/L NaClO_4_, *s*/*l* = 0.6 g/L).

Fig 4 shows the sorption edge of U(VI) on SONPs as a function of pH at different U(VI) concentrations. It can be seen that, at pH < 3.5, U(VI) sorption was quite low, while a sharp increase was observed from ~15% to ~100% as the pH increased from 3.5 to 6.0. With the increasing pH, the silanol group (= SiOH) on SONPs surface was gradually deprotonated to negatively charged (= SiO^-^) (S2 Fig), which can easily form complexes with the positively charged U(VI) species, such as UO_2_^2+^, UO_2_OH^+^ and (UO_2_)_3_(OH)_5_^+^ [2,3,9]. A significant decline of U(VI) sorption was observed above pH 9.0, which was possibly contributed to increasing electrostatic repulsion between the negatively charged SONPs surface (pH_pzc_~7.0) and U(VI) species like UO_2_(OH)_3_^-^, (UO_2_)_2_(CO_3_)_2_^2-^ and (UO_2_)_2_(CO_3_)_3_^4-^ [3,31]. Another possibility is that the dissolution of SONPs at strong alkaline condition can reduce the sorption of U(VI) to some extent. With the increases in pH, the drop of U(VI) sorption indeed reached about 30%, however it seems not consistent with the negligible dissolution of SONPs in a wide pH region (Data not shown) [16]. Therefore, the increasing electrostatic repulsion effect is the dominant driving force to the decline of U(VI) sorption rather than the dissolution of SONPs. There was a similar trend observed as well for the sorption of U(VI) on attapulgite and quartz [2,32]. At higher initial U(VI) concentrations (2.0×10^−5^ and 2.0×10^−4^ mol/L), the sorption edge shifted to higher pH with more than 0.5 pH units, which was in accordance with U(VI) uptake on montmorillonite [32].

### Effect of Ionic Strength

**Fig 5:**
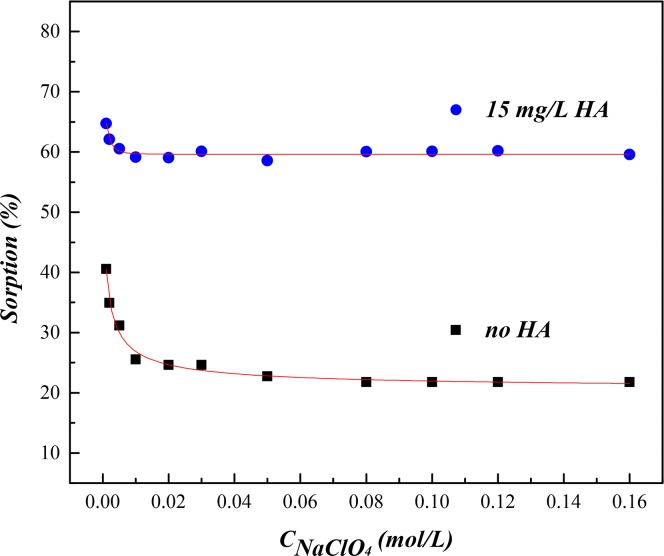
The influence of ionic strength on U(VI) sorption to SONPs (*T* = 298±1 K, s/l = 0.6 g/L, [U(VI)] = 2.0×10^−5^ mol/L, *pH* = 4.7±0.1).

Ionic strength is another important factor affecting the interaction, especially the sorption behaviors of pollutants in the environment. The effect of NaClO_4_ concentration on U(VI) sorption in the binary SONPs/U(VI) and the ternary SONPs/U(VI)/HA systems is presented at pH 4.7 in Fig 5, where the sequestration of U(VI) was controlled by the positively charged U(VI) species. At a low NaClO_4_ concentration (below 0.04 mol/L), the U(VI) sorption gradually decreased by approximately ten percent and then maintained a constant level as NaClO_4_ concentration increased, and a similar phenomenon was also observed in the ternary SONPs/U(VI)/HA system. Due to the coupling effects of ion competition, the slow decrease in U(VI) sorption was mainly caused by the competition of Na^+^. This results indicated that U(VI) sorption on SONPs was controlled by the outer-sphere complexes (OSCs) in low pH range [2]. In this case, the competition effect could be restricted to a large extent by the addition of HA, especially at the acidic condition. Fig 5 showed that 15 mg/L HA can enhance the U(VI) sorption on SONPs to a large extent compared with that in the binary SONPs/U(VI) system, which can be possibly attributed to the ternary surface complexes of SONPs-HA-U(VI) (Type A Complexes). As expected, in the presence of HA, the extent of decreased U(VI) sorption clearly became much lower as ionic strength increased in comparison with the binary SONPs/U(VI) system. The results suggested that the OSCs were the main sorption mechanism. However, the stronger ternary surface complexes, i.e., Type A Complexes, was formed in the presence of HA [3,33,34]. The effect of HA on U(VI) sorption will be further discussed in the following section.

### Effect of HA

**Fig 6:**
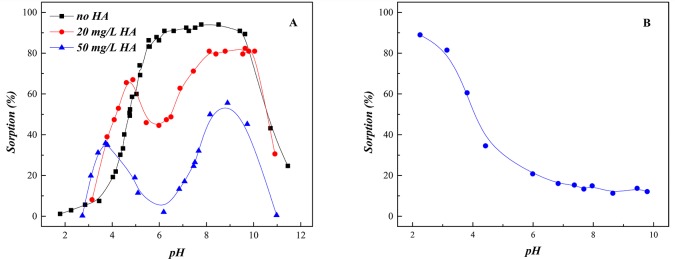
The influence of HA on the interaction between U(VI) and SONPs (*T* = 298±1 K, *I* = 0.01 mol/L NaClO_4_, *s*/*l* = 0.6 g/L). (A) The influence of HA on U(VI) sorption edge on **SONPs** ([U(VI)] = 2.0×10^−5^ mol/L). (B) HA sorption edge on **SONPs ([**HA] = 20 mg/L).

The major, often dominant, colloid-particle NOMs (*e*.*g*., fulvic and humic acids, and humin) can affect the mobility, migration, bioavailability and toxicity of metal ions in the environment [34]. Thus, the effect of HA on U(VI) sorption edge was estimated and shown in Fig 6A. In the ternary SONPs/U(VI)/HA system, U(VI) sorption was obviously enhanced below pH 4.5 and then inhibited to a large extent in a pH range of 4.5–6.0. From pH 6.0 to pH 9.0, the sorption of U(VI) increased again and then finally decreased with the increasing pH, which was basically similar to the binary SONPs/U(VI) system. As shown in Fig 6A, the trend of U(VI) sorption edge was completely changed in the ternary SONPs/U(VI)/HA system in comparison with that in the HA-free system, which indicated that the sorption mechanism of U(VI) on SONPs was changed a lot.

Fig 6B shows that HA sorption on SONPs quickly decreased from 90% to 10% as the pH increased. At a low pH range, HA favorably bound to SONPs, which can supply much more function groups, such as carboxyl, phenolic, and amino, which can form Type A Complexes mentioned above. Another possibility was that the presence of HA can reduce the electrostatic repulsion between UO_2_^+^ and HA/SONPs hybrids [35–37]. Therefore, U(VI) sorption increased to some extent in the presence of HA. As pH increased, HA remaining in aqueous phase could form soluble complexes with U(VI), where HA played the role of a competitor to SONPs, and then the sorption of U(VI) was reasonably inhibited somewhat [37].

Above pH 6.0, U(VI) sorption gradually raised again in the ternary SONPs/U(VI)/HA system, but did not reach the level in the absence of HA (Fig 6A). This difference was possibly attributed to the prevalent humate-U(VI) complexes, i.e., (UO_2_OH)^+^-HA, which firmed up U(VI) sorption. Around pH 9.0, the sorption percentage declined rapidly with a trend similar to that of the HA-free system because the concentration of dissolved carbonate and uranyl–carbonate complexes increased with the increasing pH [38]. Fan et al. [39] and Li et al. [9] also found that U(VI) sorption was obviously reduced in the alkaline pH region. In addition, it is expected that the inhabitation/enhancement of sorption became more evident in a high HA content. This result is in accordance with U(VI) sorption on oxides, carbon nanotubes and clay minerals in the presence of HA [9,40,41].

**Fig 7:**
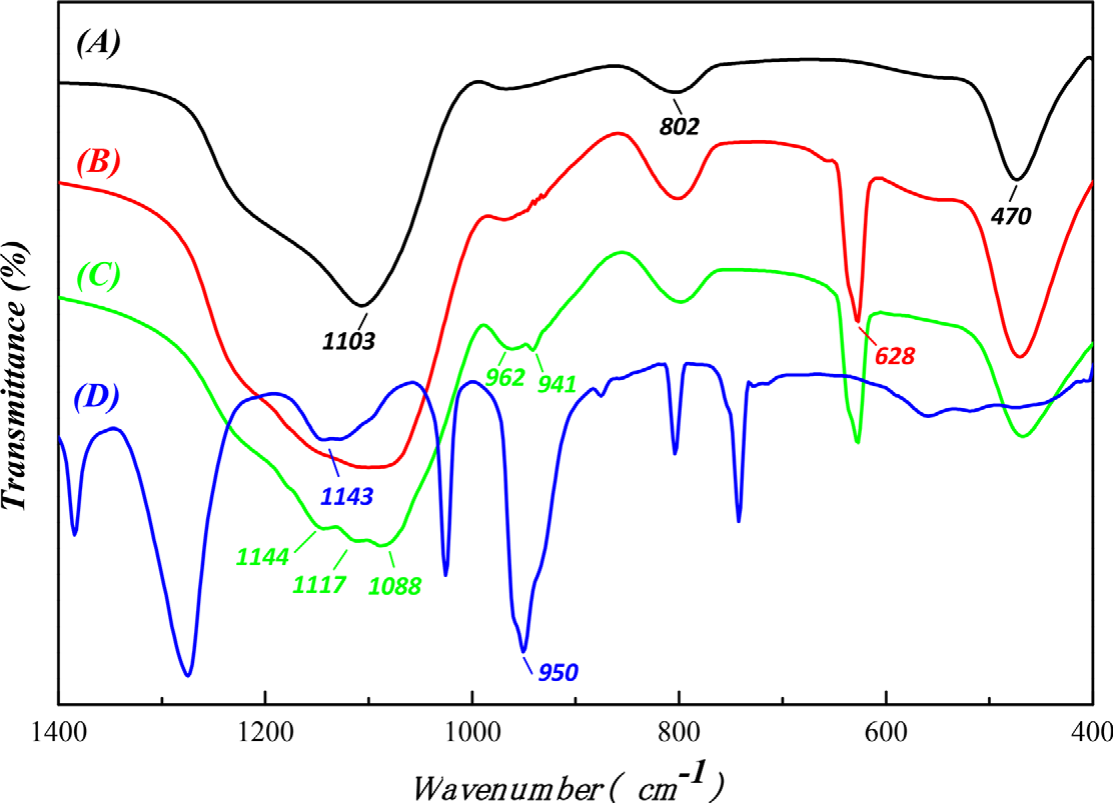
The FT-IR spectrums of unitary/binary/ternary complexes. (A) **SONPs.** (B) **SONPs-HA (*pH* = 3.0).** (C) **SONPs-HA-U(VI) (*pH* = 3.0). (D) UO**_**2**_**(NO**_**3**_**)**_**2**_**.**

To identify the molecular structure of the chemical groups on SONPs in acidic conditions, FT-IR spectroscopy was applied (Fig 7). For SONPs, the absorption peaks at 1103, 802, and 470 cm^−1^ were attributed to asymmetric and symmetric stretching vibrations and the bending vibration of Si-O-Si, respectively [23]. In the presence of HA, a peak at 628 cm^-1^ appearing in the HA/SONPs hybrids could be ascribed to the out-of-plane bending vibration of the aromatic C-H of the HA adsorbed on SONPs. It is interesting to notice that, in the ternary SONPs/U(VI)/HA system, a doublet at 962 and 941 cm^-1^ appeared (Fig 7C), which originated from the asymmetric stretching of UO_2_^2+^ [11]. Moreover, the location of the broader doublet (962 and 941 cm^-1^), the bands and the shoulders in the region of 1000–1200 cm^-1^ could possibly indicate that the strong ternary Type A Complexes formed, presenting as SONPs-HA-U(VI), which also coincided with those of the asymmetric stretching (950 cm^-1^) and the bending vibration (1143 cm^-1^) of UO_2_^2+^ (Fig 7D) [4,35,42]. The spectroscopic analysis suggested that HA did affect the interaction between the nano-pollutant and U(VI).

### Thermodynamic Estimation

**Fig 8:**
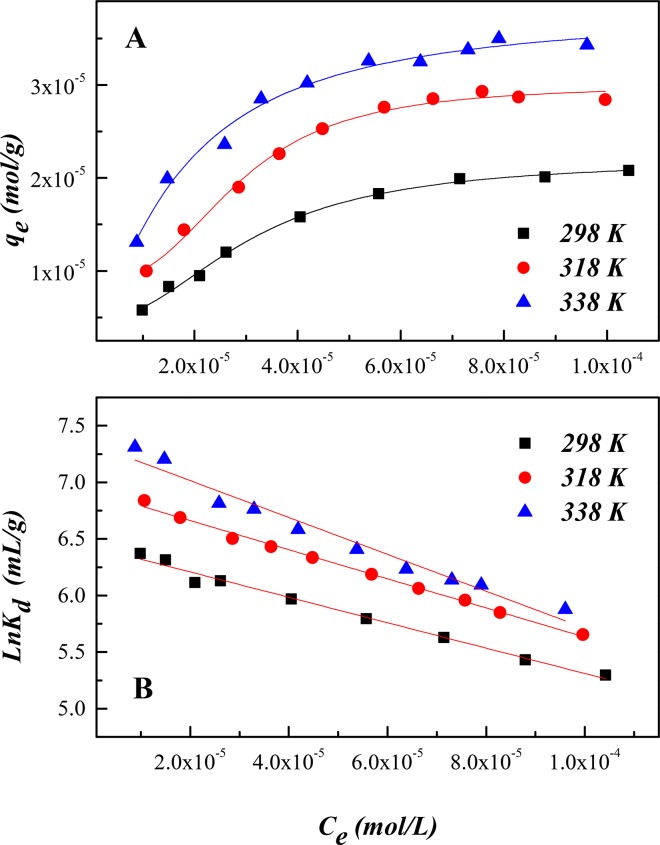
The thermodynamics of U(VI) sorption on SONPs (*I* = 0.01 mol/L NaClO_4_, *s*/*l* = 0.6 g/L, *pH* = 5.0±0.1). **(A) Sorption isotherms at three different temperatures. (B)** Linear plots of ln*K*_*d*_ versus *C*_*e*_**.**

U(VI) sorption on SONPs with respect to the temperature effect are demonstrated in Fig 8A. The enhanced sorption of U(VI) with an increasing temperature indicated that U(VI) sorption was endothermic. Similar observations have been reported for U(VI)/iron oxyhydroxide and U(VI)/granite systems [9,41]. Moreover, temperature may have two distinct effects on a chemical reaction: (i) the rate of approaching equilibrium and (ii) the position/state of equilibrium [42].

For modeling the sorption isotherms, Langmuir, Freundlich, Temkin, and Dubinin–Radushkevich (D–R) models were employed (S3 Fig), and the relative parameters are presented in Table 2. The Langmuir model, describing homogeneous sorption and predicting a single maximum binding capacity [25], fitted the sorption process better than the others. It was probable that the monolayer sorption was prevalent for U(VI) sorption on SONPs [39]. Temkin isotherm assumes that: (i) the heat of sorption of all the molecules in the layer decreases linearly with coverage due to adsorbate-adsorbent interactions, and (ii) sorption is characterized by a uniform distribution of binding energy, up to some maximum binding energy [29]. *K*_T_ (L/g) and *B* (kJ/mol), the equilibrium-binding constant and the heat of sorption, respectively, are presented in Table 2.

**Table 2:** Relative Parameters of Langmuir, Freundlich, Temkin and D-R Models of U(VI) Sorption on SONPs.

**Models**	**Parameters**	**Temperature**		
		298 K	318 K	338 K
***Langmuir model*** (Langmuir, 1918)	*q*_max_ (mol/g)	2.92×10^−5^	3.83×10^−5^	4.15×10^−5^
$\frac{C_{e}}{q_{e}}=\frac{1}{K_{L}q_{\text{max}}}+\frac{C_{e}}{q_{\text{max}}}$	*K*_L_ (L/g)	2.63×10^4^	3.72×10^4^	5.91×10^4^
	*R*^2^	0.9855	0.9780	0.9943
***Freundlich model*** (Freundlich, 1906)	*K*_*F*_ (mol^1−n^L^n^/g)	3.64×10^−3^	2.98×10^−3^	1.42×10^−3^
log *q*_*e*_ = log *K*_*F*_ + *n* log *C*_*e*_	n	0.55	0.49	0.39
	*R*^2^	0.9560	0.9283	0.9260
***Temkin model*** (Temkin and Pyzhev, 1940)	*K*_*T*_ (L/g)	0.99	0.99	0.99
*q*_*e*_ = *B* ln *K*_*T*_ + *B* ln *C*_*e*_	*B* (kJ/mol)	14.27	10.31	10.51
	*R*^2^	0.9808	0.9542	0.9637
***D-R model*** (Dubinin et al., 1947)	*Q*_*DR*_ (mol/g)	7.7×10^−6^	1.7×10^−5^	2.3×10^−5^
ln *q*_*e*_ = ln *Q*_*DR*_ − *K*_*DR*_*ε*^2^	*K*_*DR*_ (mol^2^/kJ^2^)	0.47	0.51	0.49
	*E* (kJ/mol)	1.02	0.99	1.01
	*R*^2^	0.6756	0.9771	0.9037

The thermodynamic parameters (Δ*H*^0^, Δ*S*^0^ and Δ*G*^0^) for U(VI) sorption on SONPs are calculated from the temperature-dependent sorption isotherms. The Gibbs free energy change (Δ*G*^0^) is calculated by the following equation:

$$\Delta G^{0}=-RT\;\text{ln}\;K^{0}$$ (2)

where *K*^0^ is the sorption equilibrium constant. Values of ln*K*^0^ are the intercepts obtained by plotting ln*K*_d_ versus *C*_e_ (Fig 8B). The standard entropy change (Δ*S*^0^) and enthalpy (Δ*H*^0^) were calculated using the following equations:

$$\Delta S^{0}=-{\left(\frac{\partial \Delta G^{0}}{\partial T}\right)}_{p}$$ (3)

$$\Delta G^{0}=\Delta H^{0}-T\Delta S^{0}$$ (4)

The values obtained from Eqs (2)–(4) are tabulated in Table 3. The positive values of Δ*H*^0^ indicated that the sorption was endothermic in nature. The negative Δ*G*^0^ values, as expected, could infer that the sorption is a spontaneous process under the experimental conditions. As the temperature increased, the decreasing values of Δ*G*^0^ indicated that the sorption became more efficient.

**Table 3:** Thermodynamic Parameters for U(VI) Sorption on SONPs.

Temperature	*ΔH*^*0*^ (kJ/mol)	*ΔG*^*0*^ (kJ/mol)	*ΔS*^*0*^ (J/(mol∙K))
298 K	19.02	-15.94	
318 K	19.01	-17.14	117.32
338 K	19.02	-18.19	

### Reversibility of the Interactions between U(VI) and SONPs

Sorption-desorption evaluation was adapted to study the reversibility of the interaction between U(VI) and SONPs. The sorption/desorption hysteresis coefficient *HC*%, an indirect factor to evaluate the quantity of adsorbate binding on solid phase [1,43], can be calculated as:

$$HC\%=\frac{{\bar{K}}_{d}{}_{\left(desorb\right)}-{\bar{K}}_{d}{}_{\left(sorb\right)}}{{\bar{K}}_{d}{}_{\left(desorb\right)}}\times 100\%$$ (5)

where ${\bar{K}}_{\text{d}(\text{sorb})}$ and ${\bar{K}}_{\text{d}(\text{desorb})}$ (mL/g) are the average distribution coefficient of sorption and desorption, indicating the average quantity of adsorbate in the forward-binding and backward-releasing process from the solid phase, respectively [1].

**Fig 9:**
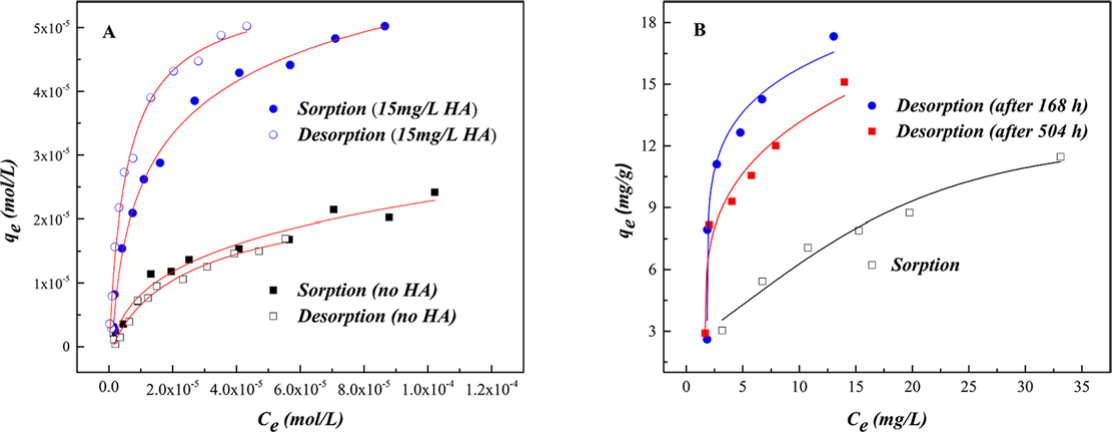
Sorption/desorption isotherms in the binary and ternary system (*T* = 298±1 K, *I* = 0.01 mol/L NaClO_4_, *s*/*l* = 0.6 g/L, *pH* = 4.5±0.1). (A) The isotherms of U(VI) on **SONPs in the binary** SONPs/U(VI) and the ternary SONPs/U(VI)/HA **system.** (B) The isotherms of HA on **SONPs in the binary** SONPs/HA **system.**

Sorption/desorption isotherms of U(VI) on SONPs are shown in Fig 9A, revealing that U(VI) sorption on SONPs is reversible, in accord with the negative value of *HC*% (Table 4), which might be related to the OSCs of U(VI) on SONPs and was also confirmed from the ionic strength-dependence in Fig 5 [44]. In contrast, the sorption of U(VI) became an irreversible process in the presence of HA, indicating that the ternary surface complexes were mainly contributed to U(VI) sorption as Type A complexes, where HA played a role of “bridge” between SONPs and U(VI) [1,43]. As shown in Table 4, the positive value of *HC*% was consistent with the irreversible process, and the values of both ${\bar{K}}_{\text{d}(\text{sorb})}$ and ${\bar{K}}_{\text{d}(\text{desorb})}$ for the ternary SONPs/U(VI)/HA system are much bigger than those for the binary SONPs/U(VI) system, which suggested that much more U(VI) was restrained in solids. Moreover, the sorption of HA on the SONPs surface should, as expected, be irreversible.

**Table 4:** The Average Distribution Coefficient Values and Sorption-desorption Hysteresis (*HC*%) of HA and U(VI) Sorption in the Binary and Ternary System.

Sorption/desorption system	${\bar{K}}_{\text{d}(\text{sorb})}$ (mL/g)	${\bar{K}}_{\text{d}(\text{desorb})}$ (mL/g)	*HC*%
The binary SONPs/U(VI) system	838	826	-1.5
The binary SONPs/HA system (*Desorption After 168 h*)	607	2646	77
The binary SONPs/HA system (*Desorption After 504 h*)	637	2086	69
The ternary SONPs/U(VI)/HA system (*15 mg/L HA*)	3272	7935	59

To confirm that the existence of HA was related to the irreversible process, the sorption/desorption experiments of HA-bound SONPs were examined (Fig 9B). It was obvious that the sorption of HA on SONPs was irreversible (in keeping with the positive value of *HC*% in Table 4), and the desorption plots were much higher than those of sorption, suggesting that stable and chemical binding resided between HA and SONPs, in particularly for the desorption after 168 h [45]. It can be speculated that surface complexes of U(VI) preferred forming Type A complexes (SONPs-HA-U(VI)) rather than U(VI) adsorbed on SONPs directly (possible complexes of HA-SONPs-U(VI) (Type B complexes) and SONPs-U(VI)) [41,46]. Moreover, the values of *K*_d(desorb)_, more than twice those of *K*_d(sorb)_ in the ternary SONPs/U(VI)/HA system with positive values of *HC*%, indicated that the adsorbate was very difficult to desorb to the aqueous phase, which was consistent with the experimental data. It can be seen that the interaction between SONPs and U(VI) would be affected by HA, whether for the sorption mechanism or the stability of the nano-contaminants, which will be discussed in the following section.

### Stability of SONPs Suspensions in Different Systems

**Fig 10:**
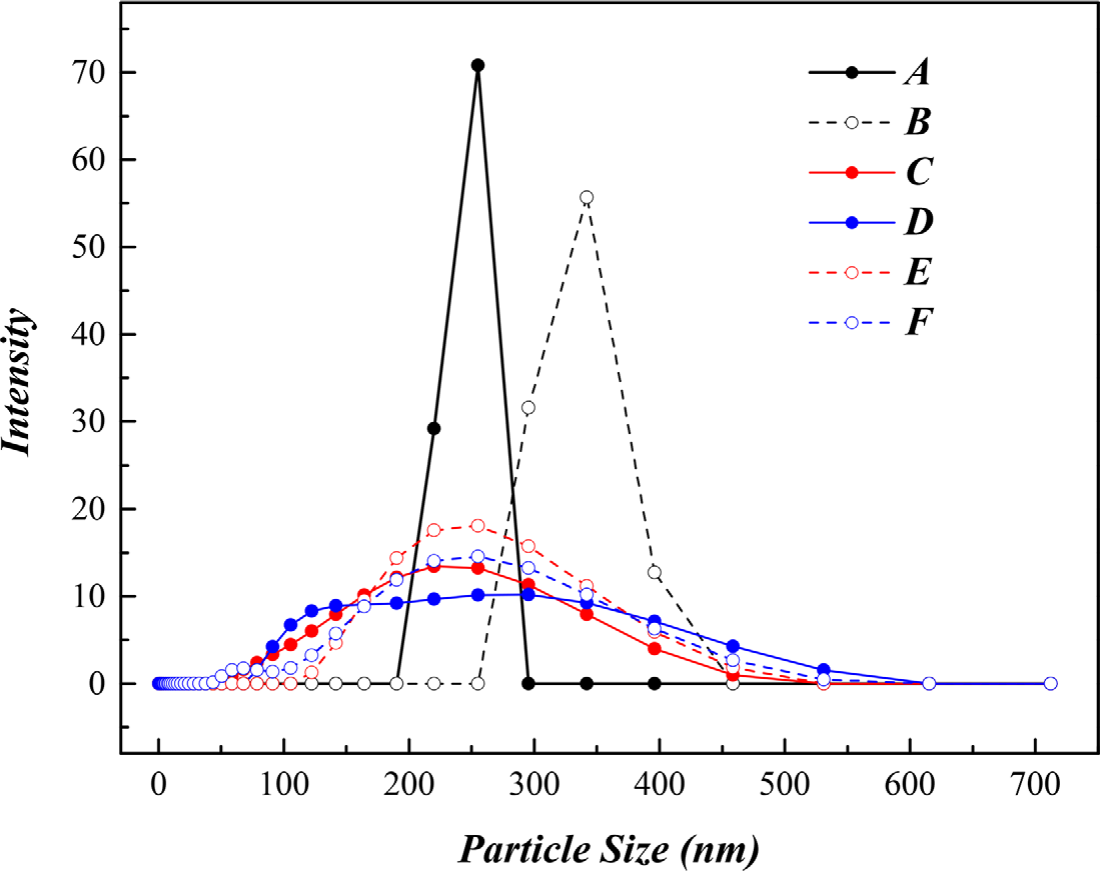
Hydrodynamic diameter (*d*_h_) number distributions presented as particle size distribution of SONPs (10 mg/L) in different systems at *pH* 4.5±0.1. (A) The unitary SONPs system. (B) The binary SONPs/U(VI) system. (C) The binary SONPs/HA system (15 mg/L HA). (D) The binary SONPs/HA system (50 mg/L HA). (E) The ternary SONPs/U(VI)/HA system (15 mg/L HA). (F) The ternary SONPs/U(VI)/HA system (50 mg/L HA).

The mobility of both SONPs and U(VI) is generally affected by one another in the absence or presence of HA, therefore their hazardous impact on the environment would change accordingly [12]. The aggregate size (hydrodynamic diameter, *d*_h_) of SONPs was examined in different system after interactions among SONPs, U(VI) and HA at pH 4.5 (Fig 10).

Compared with the size of colloidal SONPs (d_h_ = 200–300 nm), it was apparently that d_h_ increased to ~350 nm after adsorbing U(VI) (Fig 10B), which suggested that interaction between SONPs and U(VI) can effectively reduce the mobility and bioavailability of both contaminations. In the presence of HA, a relatively wide distribution of diameter observed with/without U(VI) indicated that the mobility of SONPs was not changed obviously. Moreover, a slightly strengthen intensity of particle size in the ternary SONPs/U(VI)/HA system could still account for the effect of U(VI) (Fig 10E and 10F). Similar influence of HA on nanoparticles suspension stability has been reported previously [47,48].

## Conclusions

The sorption of U(VI) on SONPs was strongly dependent upon the pH and ionic strength. The presence of HA significantly promoted U(VI) sorption under acidic conditions while inhibited in alkaline conditions. The reversible interaction of U(VI) on SONPs suggested that the OSCs controlled the interaction between U(VI) and SONPs in the observed pH range, which was ionic strength dependence. Moreover, it was very interesting that U(VI) sequestration process in the ternary SONPs/U(VI)/HA system, also the interaction between HA and SONPs, were irreversible, suggesting that HA acted as a bridge between the U(VI) and SONPs surface forming the Type A Complexes (SONPs-HA-U(VI)) rather than Type B Complexes (U(VI)-SONPs-HA) or SONPs-U(VI) complexes. FT-IR also confirmed the Type A Complexes formed in the presence of HA. Higher temperatures were found favoring U(VI) retention on SONPs.

Species description at atomic-scale, (e.g. outer- and inner-sphere complexes) will be confirmed by XAS in the future study, which could corroborate the effective sequestration of U(VI) by SONPs. The interaction of different contaminations can enhance immobilization and reduce bioavailability of both U(VI) and SONPs to some extent, indicating that the interactions among pollutants might be beneficial for the risk reduction of inorganic and organic pollutants in environment. These findings are very important to understand the interactions between the pollutants as an example of U(VI) and SONPs.

## Supporting Information

S1 Fig The XRD pattern of SONPs. (TIF)

S2 Fig The actual acid–base titration data of SONPs (*T* = 293±1 K, *I* = 0.01 mol/L NaClO_4_, [SONPs] = 5.0 g/L). (TIF)

S3 Fig Simulations of U(VI) sorption on SONPs with models (*I* = 0.01 mol/L NaClO_4_, *s/l* = 0.6 g/L, [U(VI)] = 2.0×10^−5^ mol/L, *pH* = 4.5±0.1). (A) Langmuir model. (B) Freundlich model. (C) Temkin model. (D) D–R model.
(TIF)
