# Supplementary figures and images for: Interactions between Silicon Oxide Nanoparticles (SONPs) and U(VI) Contaminations: Effects of pH, Temperature and Natural Organic Matters
Source: PLoS One. 2016 Mar 1;11(3):e0149632. doi: 10.1371/journal.pone.0149632 (PMC4773229; doi:10.1371/journal.pone.0149632)

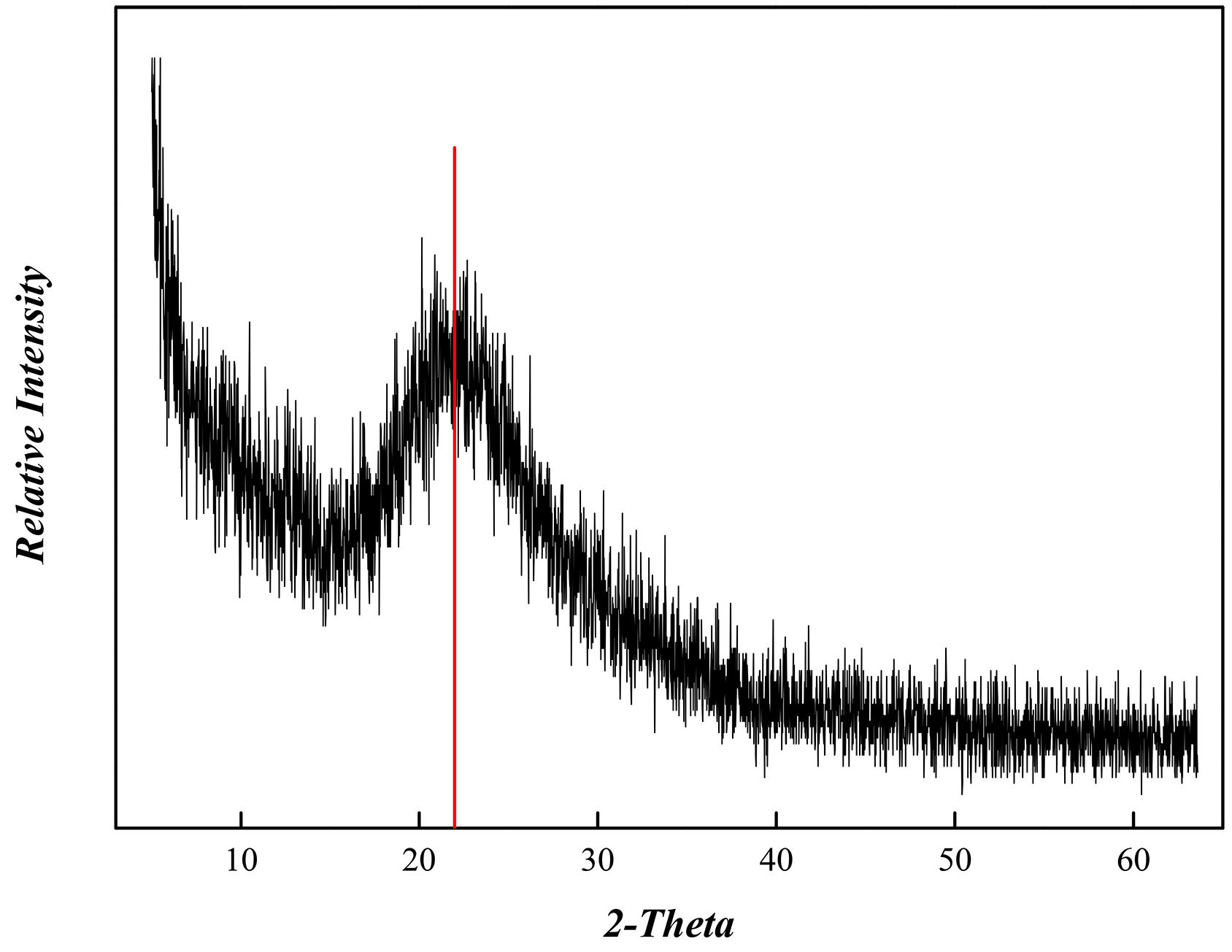

Supplement: S1 Fig — (TIF) [file pone.0149632.s001.tif]

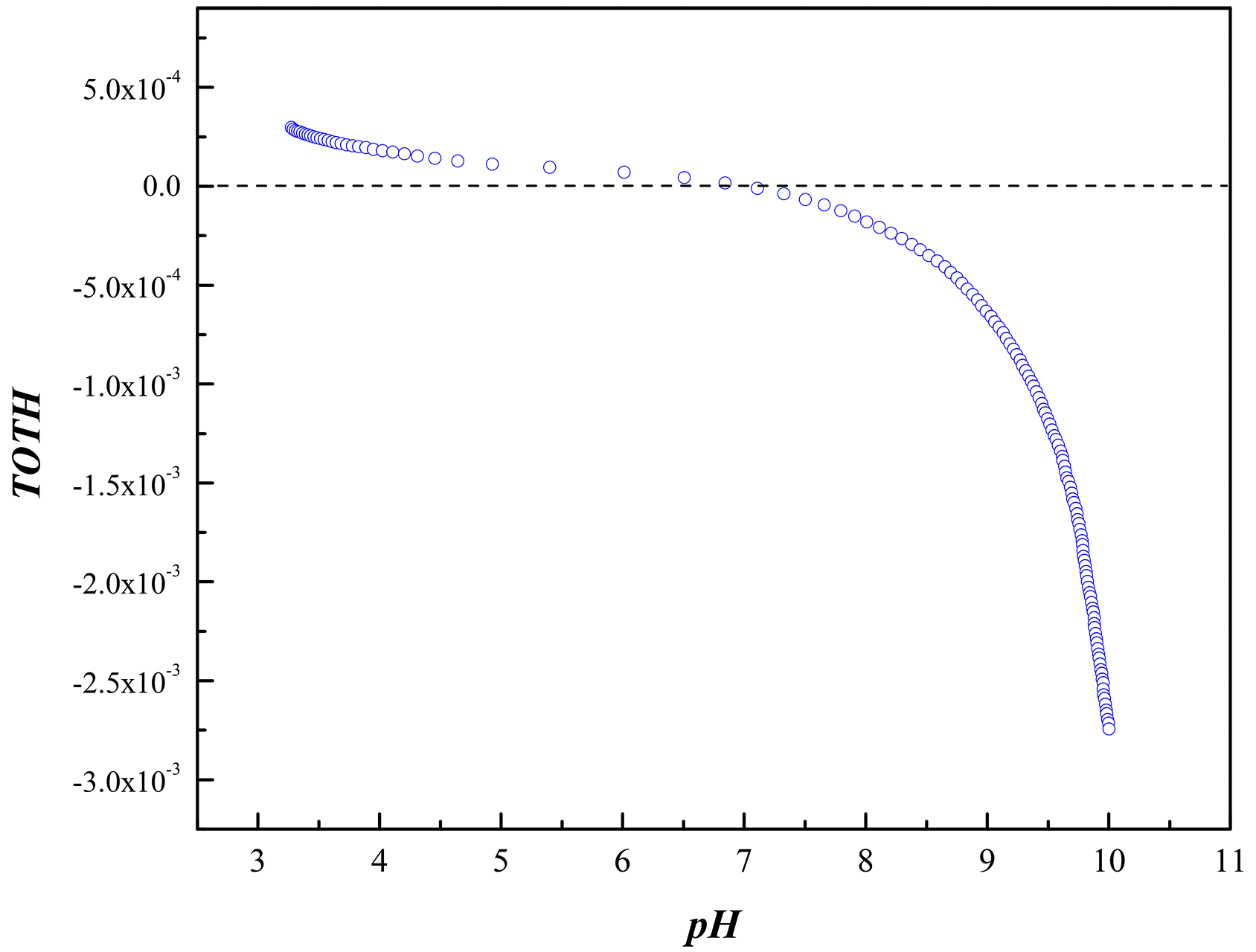

Supplement: S2 Fig — (TIF) [file pone.0149632.s002.tif]

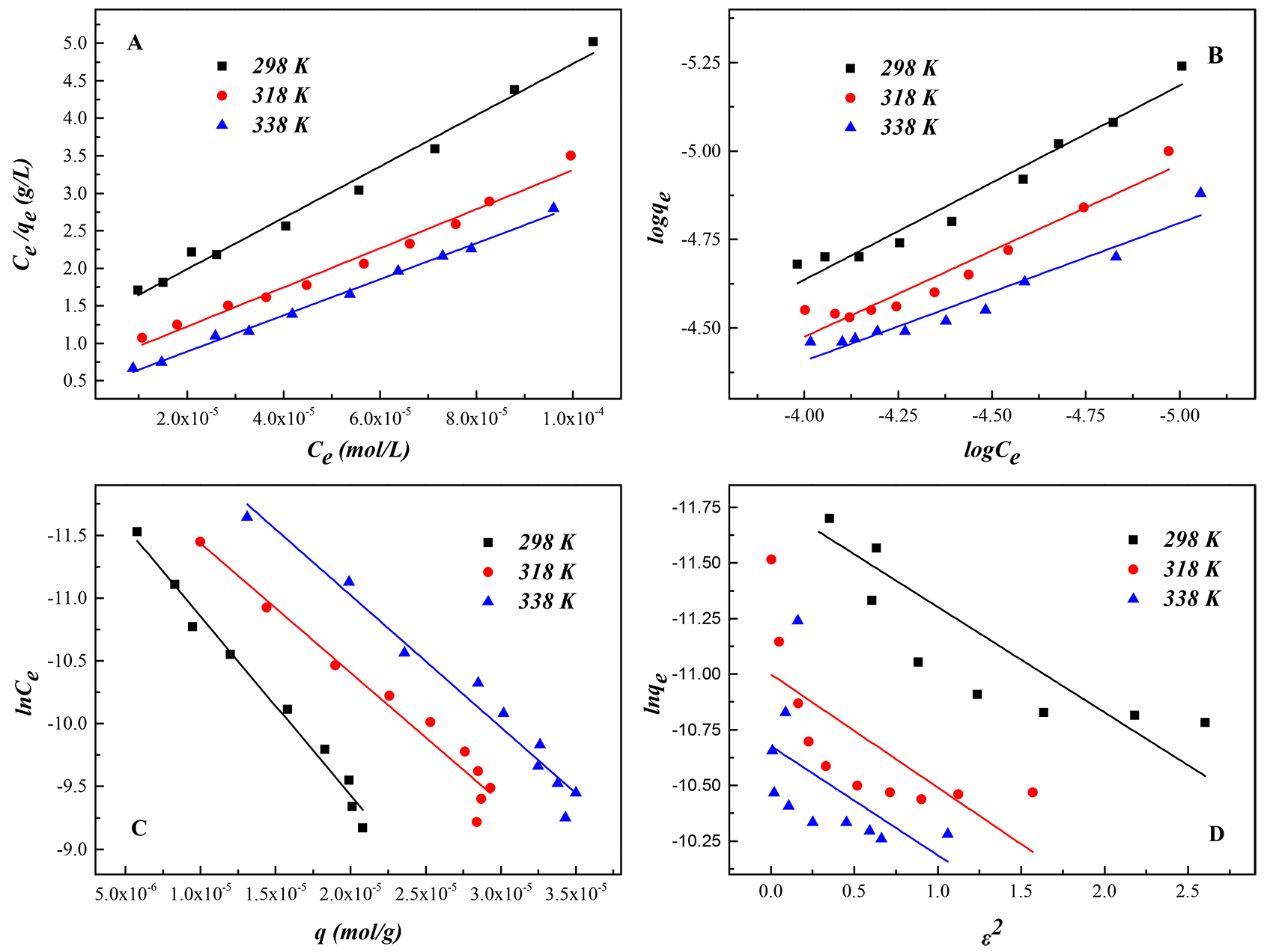

Supplement: S3 Fig — (A) Langmuir model. (B) Freundlich model. (C) Temkin model. (D) D–R model. (TIF) [file pone.0149632.s003.tif]
